# Directed Biosynthesis of Mitragynine Stereoisomers

**DOI:** 10.1021/jacs.2c13644

**Published:** 2023-02-22

**Authors:** Carsten Schotte, Yindi Jiang, Dagny Grzech, Thu-Thuy T. Dang, Larissa C. Laforest, Francisco León, Marco Mottinelli, Satya Swathi Nadakuduti, Christopher R. McCurdy, Sarah E. O’Connor

**Affiliations:** † Department of Natural Product Biosynthesis, Max Planck Institute for Chemical Ecology, Hans-Knöll-Straße 8, 07745 Jena, Germany; ‡ Plant Molecular and Cell Biology Program, University of Florida, Gainesville, Florida 32606, United States; § Department of Environmental Horticulture, University of Florida, Gainesville, Florida 32606, United States; ∥ Department of Medicinal Chemistry, College of Pharmacy, University of Florida, Gainesville, Florida 32610, United States

## Abstract

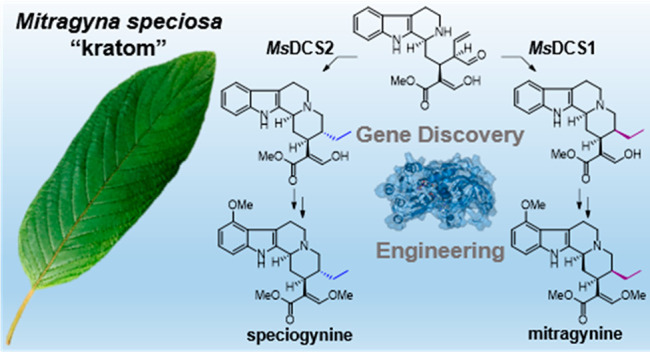

*Mitragyna speciosa* (“kratom”) is used as a natural remedy for pain and management of opioid dependence. The pharmacological properties of kratom have been linked to a complex mixture of monoterpene indole alkaloids, most notably mitragynine. Here, we report the central biosynthetic steps responsible for the scaffold formation of mitragynine and related corynanthe-type alkaloids. We illuminate the mechanistic basis by which the key stereogenic center of this scaffold is formed. These discoveries were leveraged for the enzymatic production of mitragynine, the C-20 epimer speciogynine, and fluorinated analogues.

*Mitragyna speciosa* (“kratom”) is a tree of the *Rubiaceae* family. Kratom consumption leads to stimulating effects at lower doses and opioid-like effects at higher doses.^1^ Manual workers have used it for centuries to endure heat and combat fatigue.^2,3^ Kratom is also consumed for the (self)treatment of pain, to mitigate opioid withdrawal symptoms, and to treat depression; however, rigorous clinical demonstration of kratom’s therapeutic efficacy is still lacking.^4^ Because of its purported analgesic properties, as well as for recreational purposes, kratom is increasingly used worldwide and is consumed by millions of people in the United States alone.^5,6^

The pharmacological effects of kratom have been linked to a mixture of >50 corynanthe- and oxindole-type alkaloids (Figure 1a,b).^7^ Most notable among these are the corynanthe-type alkaloid mitragynine (**1**) and the hydroxylated derivative 7OH-mitragynine (**2**). Both **1** and **2** are nanomolar partial agonists at the human μ-opioid receptor (hMOR), and **2** was found to be ∼10-fold more potent than morphine in mice.^8,9^ Intriguingly, speciogynine (**3**), the C-20 epimer of mitragynine (**1**), does not display agonist activity toward hMOR, though speciogynine (**3**), unlike mitragynine (**1**), is a smooth muscle relaxant. These differential bioactivities highlight the importance of the C-20 stereochemistry in the pharmacology of kratom alkaloids.^10^

**Figure 1:**
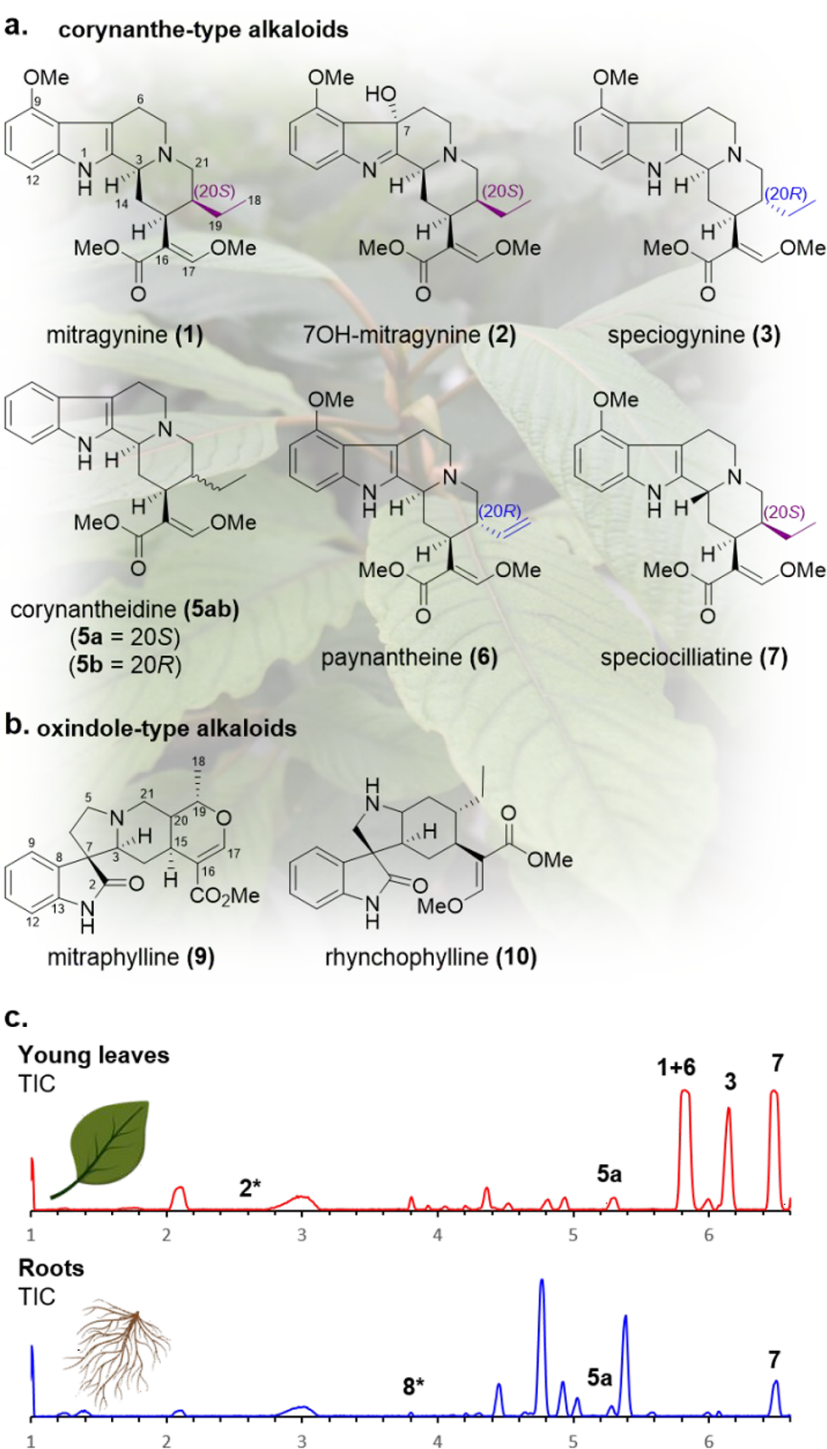
(a, b) Representative kratom alkaloids. (c) TIC of kratom leaf and root extracts. * observed in EIC.

Here, we leverage a multiomics approach to elucidate the key biosynthetic steps that form the corynanthe-type scaffold of kratom alkaloids. We report the discovery of two medium-chain alcohol dehydrogenases (*Ms*DCS1 and *Ms*DCS2) along with an enol *O*-methyltransferase (*Ms*EnolMT) that converts strictosidine aglycone (**4**) to either (20*S*)-corynantheidine (**5a**) (the precursor to **1**) or (20*R*)-corynantheidine (**5b**) (the precursor to **3**). Rational mutagenesis of *Ms*DCS1 revealed key amino acid residues that control the stereoselective reduction at C-20. A precursor directed biosynthesis approach was then used for the stereoselective production of **1** and **3**, as well as fluorinated analogues.

We first identified where these alkaloids accumulate *in planta* by analyzing methanolic extracts of *M. speciosa* root, stem, bark, and leaf tissue using targeted metabolomics (Figure 1c; Figures S1–S6). Consistent with literature reports^11,12^ the alkaloid content in the leaves was higher than other organs, with mitragynine (**1**), paynantheine (**6**), speciogynine (**3**), and speciocilliatine (**7**) being the dominant products. Low levels of 7OH-mitragynine (**2**), (20*S*)-corynantheidine (**5a**), and strictosidine (**8**) were also observed. Stem and bark showed similar metabolic profiles, with **7** as the dominant alkaloid and low quantities of **1** and **3** also observed. Notably, root tissue was completely lacking in **1** and **3**, with only **7**, **8**, and **5a** detected.

Strictosidine aglycone (**4**) is the central intermediate for most monoterpene indole alkaloids, including **1**, **3**, and other kratom-derived alkaloids. The biosynthetic pathway for **4** has been elucidated in *Catharanthus roseus*,^13^ and we identified orthologues of these biosynthetic genes in the kratom transcriptome (Scheme 2a). Notably, although **1** and **3** accumulate primarily in leaf and stem, the strictosidine aglycone (**4**) biosynthetic genes were preferentially expressed in roots, suggesting that this organ is the primary site of biosynthesis for the early pathway steps toward **1**. Therefore, either **1** and **3** are produced in the root and subsequently transported to leaf/stem, or alternatively, a biosynthetic intermediate of **1** and **3** is transported to the leaf/stem where the final biosynthetic steps would take place.

Strictosidine aglycone (**4**) is a reactive intermediate that can be reductively trapped into numerous isomers.^14,15^ One isomer, dihydrocorynantheine (**11ab**), has the same scaffold as **1** and **3**, and is therefore a likely biosynthetic intermediate for these alkaloids. Recently, we reported the discovery of a medium-chain alcohol dehydrogenase from *Cinchona pubescens*, dihydrocorynantheine synthase (*Cp*DCS), that converts strictosidine aglycone (**4**) to (20*R*)-dihydrocorynantheine (**11b**) during the biosynthesis of quinine (**12**) (Scheme 1a).^16^ The structure of **11b** was inferred based on (HR)MS/MS experiments and by NMR characterization of the decarboxylated product (20*R*)-dihydrocorynantheal (Figure S7).^16^ It seemed logical that orthologous enzymes should catalyze the reduction of strictosidine aglycone to the dihydrocorynantheine scaffold in kratom (Scheme 1b).^16,17^ Moreover, given the presence of dihydrocorynantheine-like alkaloids with both (20*S*)- and (20*R*)-stereochemistry in kratom, we further hypothesized that kratom would have multiple DCS orthologues with differing stereoselectivity.

**Scheme 1:**
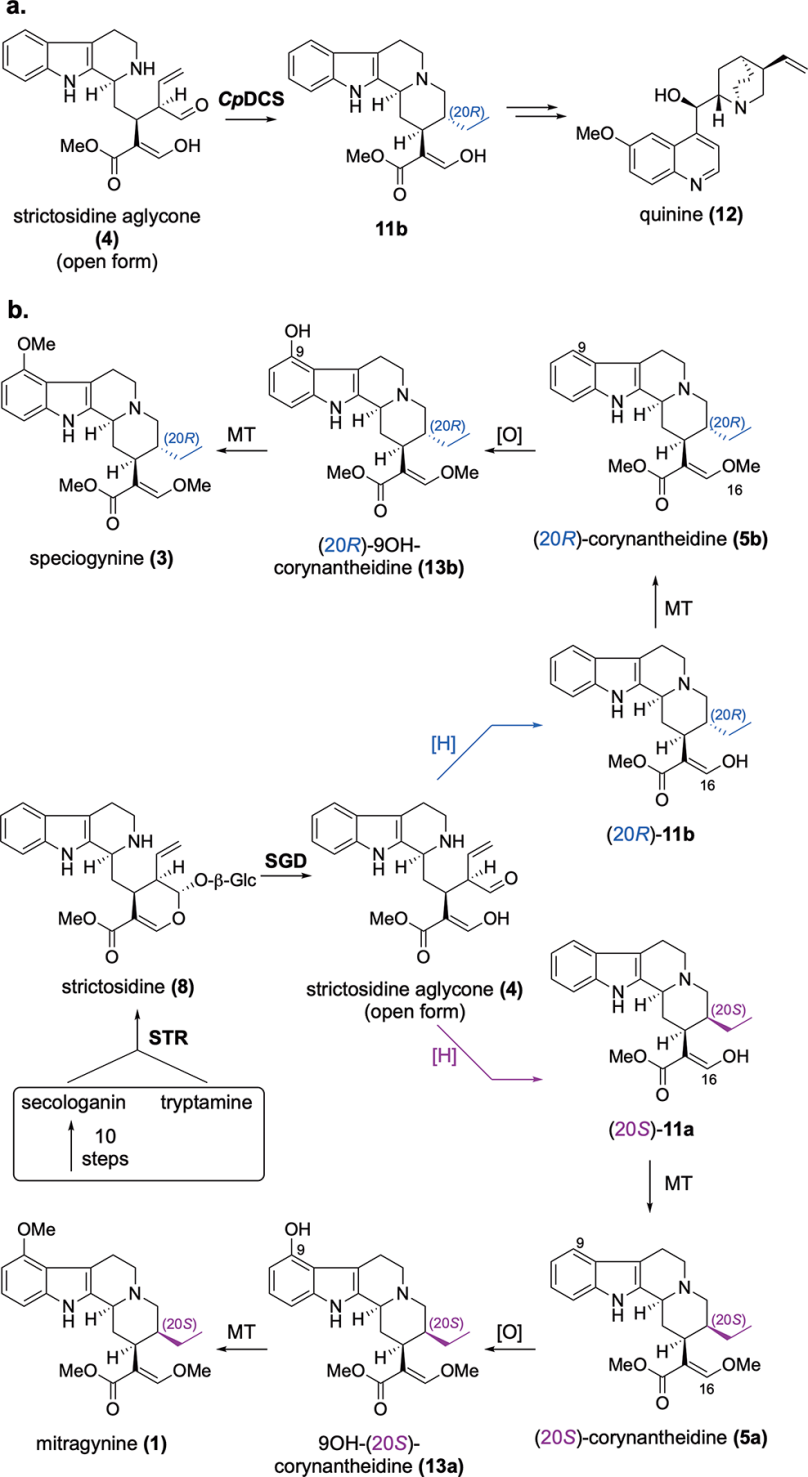
(a) Reduction of **4** by *Cp*DCS and (b) Proposed Pathway Toward Major Kratom Alkaloids

To identify enzyme candidates from kratom that catalyze formation of **11a** or **11b** from strictosidine aglycone (**4**), we used the protein sequence of *Cp*DCS to mine the kratom transcriptome. From this process, 27 candidates that showed homology to *Cp*DCS and/or coexpressed with genes involved in (**8**)-formation were expressed in *Escherichia coli* (Figures S8 and S9). To assay for enzymatic activity, strictosidine (**8**) was deglycosylated *in situ* with strictosidine glucosidase from *C. roseus* (*Cr*SGD) and incubated with kratom reductase candidates and NADPH. *Cp*DCS, which afforded (20*R*)-dihydrocorynantheine (**11b**),^16^ was used as a positive control (Scheme 2c; *T*_R_ = 4.4 min; [M + H]^+^ calcd for C_21_H_27_N_2_O_3_, 355.2022; found, 355.2014).^16^ Two of the tested kratom candidates, *Ms*DCS1 and *Ms*DCS2, also produced **11b** (Scheme 2c; Figure S10). Intriguingly, *Ms*DCS1 also yielded a second product with the same HRMS ([M + H]^+^ calcd for C_21_H_27_N_2_O_3_, 355.2022; found, 355.2015) and MS/MS fragmentation pattern as **11b**, but a different retention time (*T*_R_ = 3.9 min; Scheme 2c; Figure S10). We assumed that this product was (20*S*)-dihydrocorynantheine (**11a**), but due to poor stability, this compound could not be characterized.

**Scheme 2:**
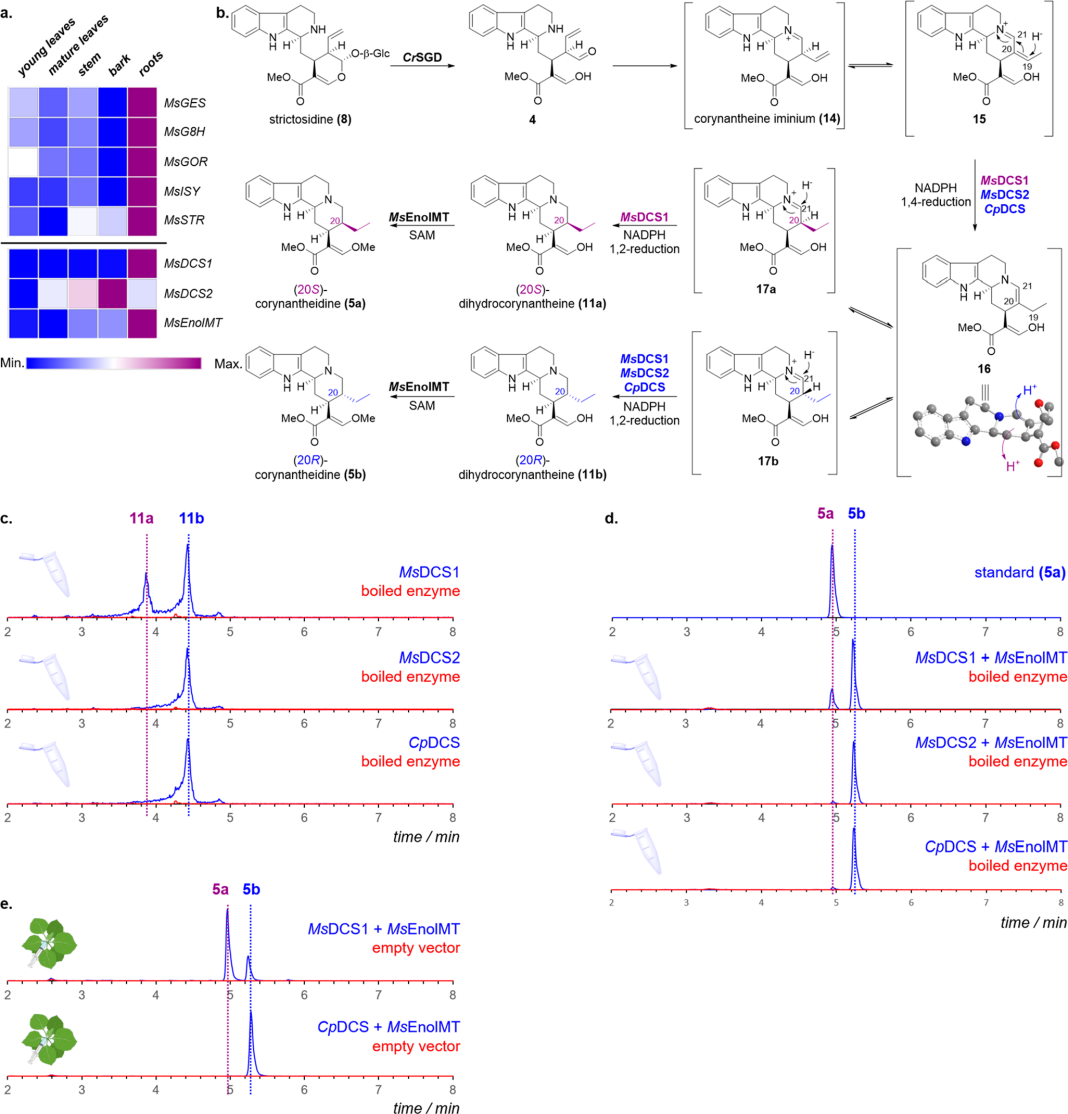
(a) Expression Profiles of Identified Genes in Kratom; (b) Proposed Mechanism for the Formation of the Corynanthe-Type Skeleton; (c) EIC (*m/z* = 355) of Assays Featuring Combinations of **8**, *Cr*SGD, and *Ms*DCS1/*Ms*DCS2/*Cp*DCS; (d) EIC (*m/z* = 369) of Assays Featuring Combinations of **8**, *Cr*SGD, *Ms*EnolMT, and *Ms*DCS1/*Ms*DCS2/*Cp*DCS; and (e) EIC (*m/z* 369) Corresponding to Transient Expression of *Cr*STR, *Cr*SGD, *Ms*DCS1/*Cp*DCS, and *Ms*EnolMT in Tobacco Expression levels are represented as FPKM of the *M. speciosa* transcriptome.

Subsequent *O*-methylation at C-16 of **11ab** would yield corynantheidine (**5ab**), which is the next predicted intermediate in the biosynthesis of **1** and **3**. To identify gene candidates that catalyze *O*-methylation at C-16 of **11ab**, we identified annotated methyltransferase genes that coexpress with *Ms*DCS1 (*r* > 0.8, Pearson correlation coefficient). Five purified enzyme candidates were assayed *in vitro* with strictosidine (**8**), strictosidine glucosidase (*Cr*SGD), NADPH, SAM, and either *Cp*DCS, *Ms*DCS1, or *Ms*DCS2. A single enzyme, *Ms*EnolMT (*r* = 0.96), showed methyltransferase activity in all enzyme assays (Scheme 2d). Coincubation of *Ms*EnolMT with *Ms*DCS1 afforded two products with the expected nominal mass of 368 corresponding to the methylated product of **11ab** (HRMS: [M + H]^+^ calcd for C_22_H_28_N_2_O_3_, 369.2178; found, 369.2164 and 369.2167). The minor product (*T*_R_ = 4.9 min) eluted at the same retention time and displayed identical MS/MS spectra compared to an authentic standard of **5a** (Figures S11 and S12),^18^ validating that *Ms*DCS1 generates (20*S*)-dihydrocorynantheine (**11a**). The major product **5b** (*T*_R_ = 5.4 min) displayed identical MS/MS patterns to **5a** (Figure S12), but different retention time. This product has the same retention time and MS/MS pattern as the product of *Cp*DCS, which had been previously established to have *R* stereoselectivity (Scheme 2c,d).^16^ Moreover, the MS/MS and the retention time of the decarboxylated major *Ms*DCS1 product matched an authentic standard of (20*R*)-dihydrocorynantheal (Figure S7).^16^*Ms*DCS1 also showed *R* stereoselectivity (Scheme 2c).

The ratios of **11a** and **11b** produced by *Ms*DCS1 varied considerably among *in vitro* assays, suggesting that assay conditions affect the stereochemical outcome. To corroborate *Ms*DCS1 activity, we transiently expressed *Ms*DCS1 together with *Cr*STR, *Cr*SGD, and *Ms*EnolMT in *Nicotiana benthamiana* leaves. Infiltration with tryptamine and secologanin (**19**) [the precursors to strictosidine (**8**)] afforded reproducible ratios of **5a** and **5b**, with **5a** as the dominant product (Scheme 2e). Exchange of *Ms*DCS1 with *Cp*DCS afforded solely **5b**, in agreement with previous observations.

Formation of **11ab** may likely proceed via an initial 1,4-reduction of the α,β-unsaturated iminium dehydrogeissoschizine (**15**), which can form *in situ* upon deglycosidation of **8** (Scheme 2b).^16^ The reduced intermediate **16** tautomerizes to the iminium form, **17ab**, upon protonation at C-20, after which a second 1,2-reduction would occur at C-21. Protonation at C-20 during tautomerization would therefore define the stereochemical outcome. We hypothesized that differences within the active site of these enzymes controlled the face of protonation.

To identify candidate amino acid residues that direct the C-20 stereochemistry, we generated a structural model of *Ms*DCS1 (Figure 2a–c).^19,20^ Seven amino acids in the binding pocket differentiate *Ms*DCS1 from *Ms*DCS2/*Cp*DCS (Figure 2c,d; Figure S13). These residues from *Ms*DCS2/*Cp*DCS were introduced into *Ms*DCS1 to swap stereoselectivity at C-20. Assays were performed by transient expression of the resulting mutants in tobacco leaves (together with *Cr*STR, *Cr*SGD, *Ms*EnolMT, tryptamine, and secologanin; Figure 2e; Figure S14). Mutagenesis of residues 295–298 (SGAS to ATGG) was sufficient to invert the ratio between **5a** and **5b** (from ∼74% **5a** in wild-type *Ms*DCS1 to <35% **5a** in the mutant). In a septuple mutant of *Ms*DCS1 (T53F, I100M, S116N, SGAS295–298ATGG), formation of the (20*S*)-isomer **5a** was nearly abolished (<5%). Similar results were observed in analogous mutations in *Cp*DCS, which forms the (20*R*) product **5b** (Figure 2f). In the septuple *Cp*DCS mutant (F53T, M100I, N116S, ATGG295–298SGAS), the amount of **5a** changed to ∼45% in the mutant compared to 0% in the wild type enzyme (Figure 2f; compare to Figure S14 for additional mutations). Mining of the kratom genome revealed that *Ms*DCS1 is the only homologue harboring these residues at these positions, so we speculate that *Ms*DCS1 is solely responsible for production of corynanthe-type alkaloids with (20*S*)-stereochemistry in kratom (Figure S15).

**Figure 2:**
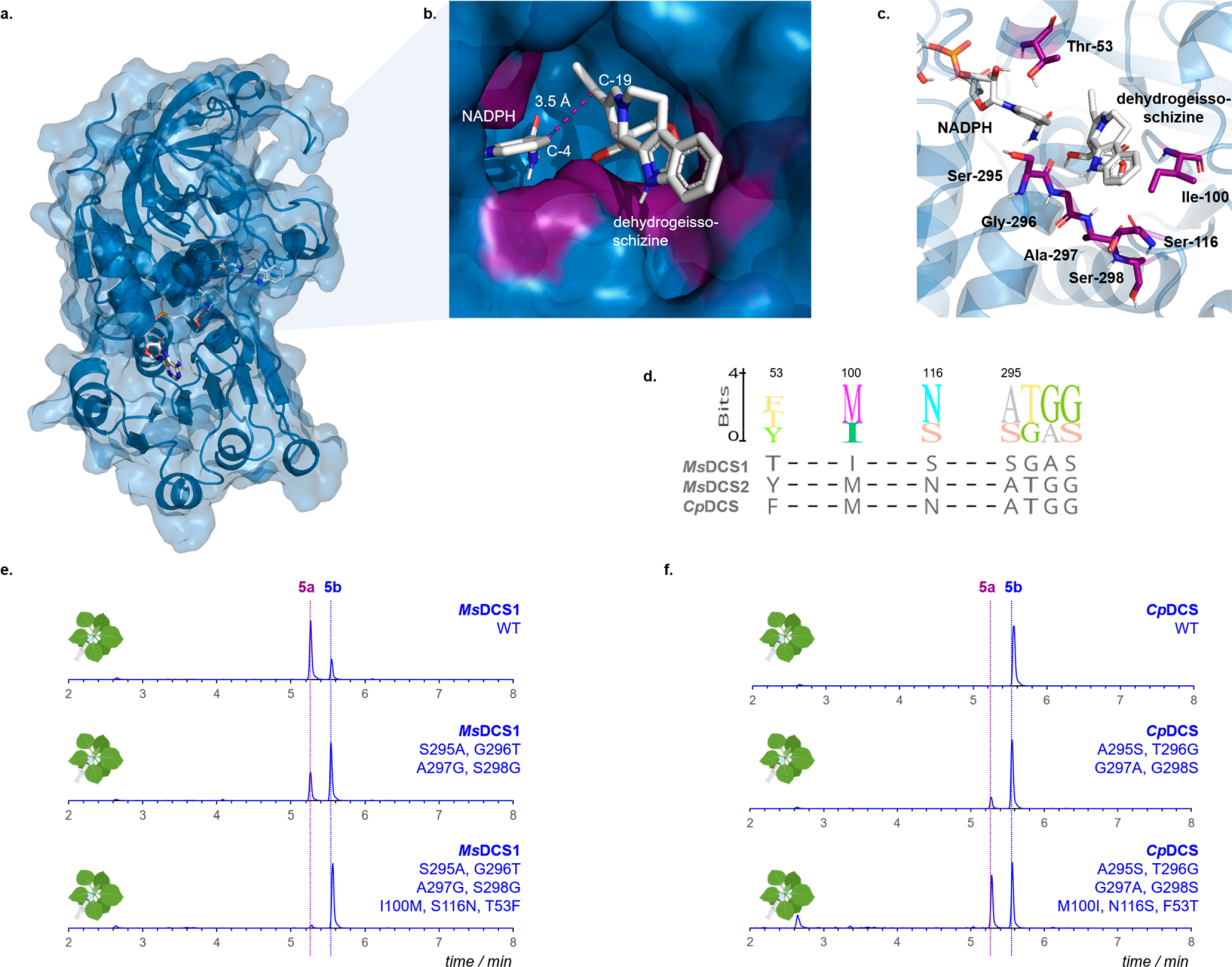
(a, b) Structural model of *Ms*DCS1 in complex with NADPH and dehydrogeissoschizine (**15**). (c) Key active site residues directing the stereoselectivity. (d) Sequence alignment between *Ms*DCS1, *Ms*DCS2, and *Cp*DCS. (e, f) Levels of (20*S*)-**5a** and (20*R*)-**5b** in mutants of *Ms*DCS1/*Cp*DCS; displayed are EICs (*m*/*z* 369) corresponding to transient expression of *Cr*STR, *Cr*SGD, and ADH mutants and *Ms*EnolMT in *Nicotiana benthamiana*.

These seven amino acids may collectively affect the orientation in which dehydrogeissoschizine (**15**) binds in the enzyme active site, which would in turn control tautomerization and protonation of **16** to either (20*S*)-**17a** or (20*R*)-**17b** (Scheme 2b; Figure S16). The model of the active site did not contain an amino acid that would be appropriately positioned to catalyze this stereoselective protonation, suggesting that a bound water molecule may be responsible, as previously proposed for other monoterpene indole alkaloid reductases.^21^ This mutational analysis lays the foundation for metabolic engineering strategies to improve production of mitragynine (**1**); for example, *Ms*DCS2 could be knocked out or mutated in kratom to generate plants with increased levels of alkaloids with (20*S*)-stereoconfiguration.

Completion of mitragynine (**1**) and speciogynine (**3**) biosynthesis requires methoxylation at C-9 of **5ab** (Scheme 1b).^22^ Since early pathway genes are expressed in roots, while **1** is found exclusively in leaves, it is difficult to predict where the genes responsible for methoxylation would be located. Therefore, we screened oxidases with a variety of expression profiles. However, although 172 candidate oxidase genes were assayed, none showed activity toward either **5a** or **5b** (Figures S17 and S18). Attempts to use the fungal cytochrome P450 monooxygenase PsiH, another oxidase known to hydroxylate this position of the indole moiety, also failed to hydroxylate **5a** or **5b** (Figure S19).^23^

Therefore, we switched to a mutasynthetic strategy to reconstitute mitragynine (**1**) biosynthesis. *N. benthamiana* leaves were transiently expressed with *Cr*STR, *Cr*SGD, *Ms*DCS1, and *Ms*EnolMT and infiltrated with 4-methoxy-tryptamine (**18**) and secologanin (**19**). Consistent with the previously observed stereoselectivity of *Ms*DCS1, this afforded a mixture of **1** and **3**, with **1** as the dominant product (Scheme 3a,b). Exchange of *Ms*DCS1 with *Cp*DCS solely afforded speciogynine (**3**). *In vitro* assays yielded similar results (Figure S20).

**Scheme 3:**
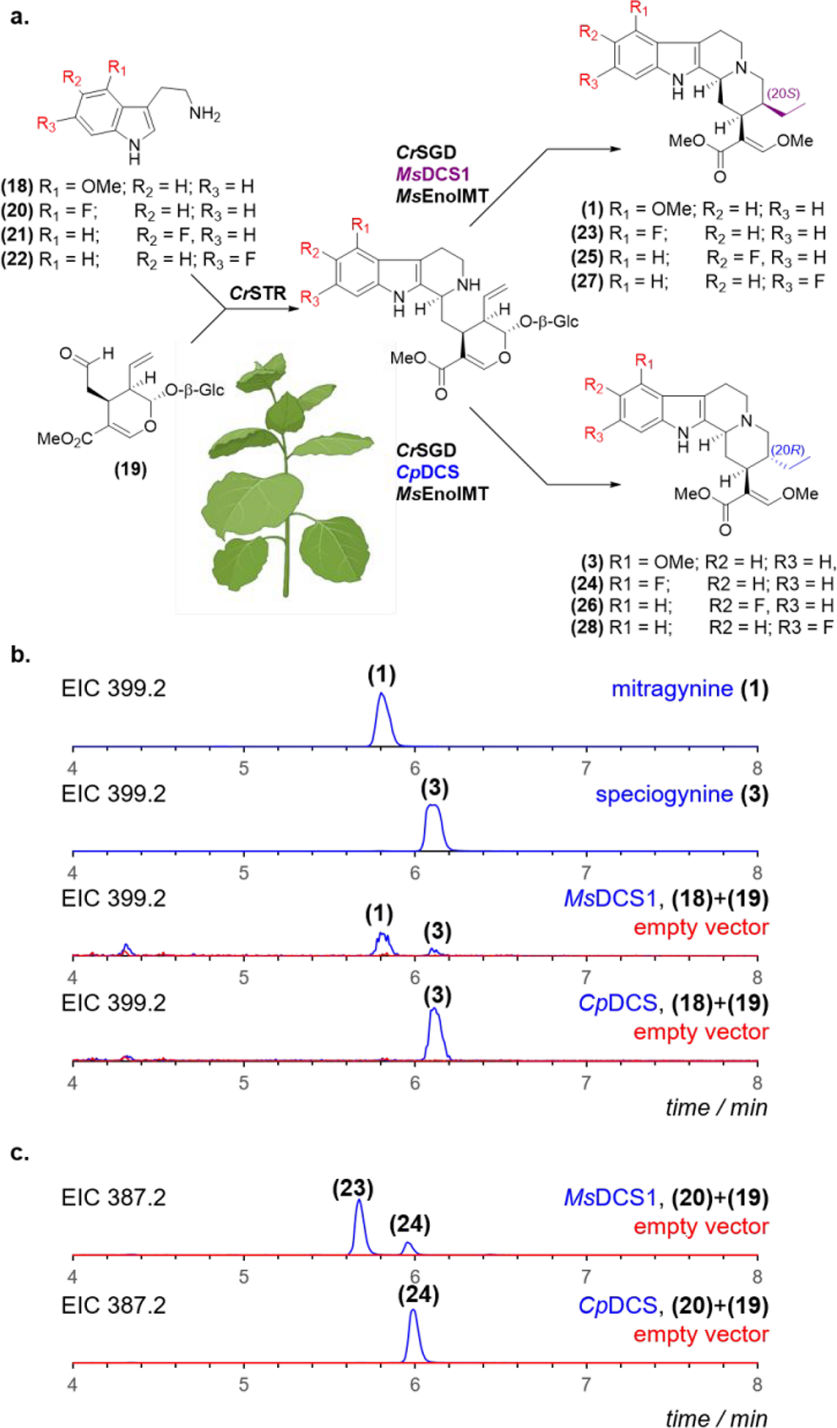
(a) *N. benthamiana* Infiltration Strategy; and (b, c) EICs of Methanolic Extracts from the Transient Expression of *Cr*STR, *Cr*SGD, *Ms*EnolMT, and Indicated ADH Enzymes with Different Tryptamine Analogues

Notably, fluorinated mitragynine analogues have enhanced pharmacological activity.^24^ Therefore, we assessed the potential for the biocatalytic production of fluorinated analogues of **1** and **3**. Infiltration of secologanin (**19**) and either 4F-, 5F-, or 6F-tryptamine (**20**–**22**), along with *Cr*STR, *Cr*SGD, *Ms*DCS1, and *Ms*EnolMT in *N. benthamiana*, afforded compounds that corresponded to the expected fluorinated analogues (**23**–**28**) as evidenced by HRMS (Scheme 3a,c; Figures S21–S26). Although attempts to isolate these compounds in quantities sufficient for NMR analysis failed, this sets the stage for exploring more efficient yeast-based strategies for mitragynine analogue engineering.

In conclusion, we elucidated the key enzymatic steps for the production of corynanthe-type alkaloids in kratom. Mutagenesis experiments suggest a mechanism that is responsible for the control of the stereochemistry at the crucial C-20 position. These discoveries will enable targeted genome editing in kratom to fine-tune alkaloid profiles. Given the recent advent of yeast expression systems for the production of monoterpene indole alkaloids,^25^ we anticipate that these enzymes will enable development of robust production platforms for mitragynine, speciogynine, and related analogues.

## Supporting Information Available

Detailed methods and materials and additional figures and data including chemical structures, LCMS results, extracted ion chromatograms, sequence alignments, pairwise sequence identities, MS/MS data, and Geneious results (PDF)

## Abbreviations

ADH: alcohol dehydrogenase
*Cr*: *Catharanthus roseus*
*Cp*: *Cinchona pubescens*
DCS: dihydrocorynantheine synthase
EIC: extracted ion chromatogram
FPKM: fragments per kilobase of exon per million of mapped fragments
GES: geraniol synthase
G8H: geraniol 8-hydroxylase
GOR: 8-hydroxygeraniol oxidoreductase
GPP: geranylpyrophosphate
ISY: iridoid synthase
hMOR: human μ-opioid receptor
HRMS: high-resolution mass spectrometry
*Ms*: *Mitragyna speciosa*
NADPH: nicotinamide adenine dinucleotide phosphate
SAM: *S*-adenosyl-methionine
SGD: strictosidine glucosidase
STR: strictosidine synthase
TIC: total ion chromatogram
